# QSRR modeling of the chromatographic retention behavior of some quinolone and sulfonamide antibacterial agents using firefly algorithm coupled to support vector machine

**DOI:** 10.1186/s13065-022-00874-2

**Published:** 2022-11-03

**Authors:** Marwa A. Fouad, Ahmed Serag, Enas H. Tolba, Manal A. El-Shal, Ahmed M. El Kerdawy

**Affiliations:** 1 Pharmaceutical Chemistry Department, Faculty of Pharmacy, Cairo University, Kasr El-Aini St, P.O. Box 11562, Cairo, Egypt; 2 Department of Pharmaceutical Chemistry, School of Pharmacy, Newgiza University (NGU), Newgiza, km 22 Cairo–Alexandria Desert Road, Cairo, Egypt; 3 Pharmaceutical Analytical Chemistry Department, Faculty of Pharmacy, Al-Azhar University, 11751 Cairo, Egypt; 4 Egyptian Drug Authority (Former National Organization for Drug Control and Research), Cairo, Egypt

**Keywords:** Quinolones, Sulfonamides, Quantitative structure-retention relationship, Firefly algorithm, Support vector machine, Y-randomization

## Abstract

Quinolone and sulfonamide are two classes of antibacterial agents with an opulent history of medicinal chemistry features that contribute to their bacterial spectrum, efficacy, pharmacokinetics, and adverse effect profiles. The urgent need for their use, combined with the escalating rate of their resistance, necessitates the development of suitable analytical methods that accelerate and facilitate their analysis. In this study, the advanced firefly algorithm (FFA) coupled with support vector regression (SVR) was used to select the most significant descriptors and to construct two quantitative structure-retention relationship (QSRR) models using a series of 11 selected quinolone and 13 sulfonamide drugs, respectively, to predict their retention behavior in HPLC. Precisely, the effect of the pH value and acetonitrile composition in the mobile phase on the retention behavior of quinolones and sulfonamides, respectively, were studied. The obtained QSRR models performed well in both internal and external validations, demonstrating their robustness and predictive ability. Y-randomization validation demonstrated that the obtained models did not result by statistical chance. Moreover, the obtained results shed the light on the molecular features that influence the retention behavior of these two classes under the current chromatographic conditions.

**Supplementary Information:**

The online version contains supplementary material available at 10.1186/s13065-022-00874-2.

## Introduction

Antibacterial resistance is a major public health concern affecting humans worldwide, owing primarily to the uncontrolled use of such bioactive compounds, particularly in countries lacking standard treatment guidelines [1]. Among those antibacterial agents, fluoroquinolones, a fluoro substituent series derived from nalidixic acid, showed an escalating rate of resistance after dominating the therapeutic practice for some time, particularly against gram-negative pathogens [2–4]. Such classes of active compounds must be carefully monitored regarding their use and abundance in the environment. Consequently, from an analytical viewpoint, the urgent detection and analysis of these drugs become essential considering the need to develop quick, simple, economical, and accurate methods for their analysis.

A review of the literature revealed that quinolones could be determined thoroughly using high-performance liquid chromatography in various matrices, including biological fluids and tissues [5–11], milk and food of animal origin [12–17], marine products [18], honey [19], wastewater [20–22] and in many pharmaceutical formulations [23–28]. Furthermore, the relationship between the retention factors and lipophilicity of quinolones has been analyzed using RP-TLC [29–31] and HPLC human serum albumin and α1-acid glycoprotein stationary phases [32]. Additionally, Wu et al. [33] investigated the retention factors-activity relationship of some quinolones using micellar chromatography.

Moreover, sulfonamides are another class of synthetic antimicrobial agents that, unfortunately, have widespread resistance, making them infrequently used for medical interventions. However, the application of sulfonamides has expanded beyond their original indication as antimicrobial agents to other new medical uses, including anticancer, antiglaucoma, cyclooxygenase-2 (COX-2) and lipoxygenase inhibitors, anticonvulsant, and hypoglycemic activities [34]. Regarding the analytical tools used in their detection, a review of the literature revealed that reversed-phase liquid chromatography was also dominant in this class’s determination [35–38]. In the context of their retention mechanisms, Cazenave-Gassiot et al., [39] studied the correlation between the sulfonamides’ retention factors and the proportion of the organic modifier in the mobile phase using supercritical fluid chromatography. However, the separation behavior of this class on reversed-phase liquid chromatography must be investigated.

Among the various models and theories used to draw an image of the retention manner of the various analytes in reversed-phase liquid chromatography, the quantitative structure-retention relationship (QSRR) provides useful insights not only in elucidating how different the analytes perform regarding their retention but also in predicting their retention chromatographic systems relatively well [40, 41]. This relationship provides a powerful alternative to the conventional trial-and-error approach with significant improvements in experiment time and cost.

A correlation is built in these mathematical models between the chemical structures of compounds represented by their descriptors and their retention data in various chromatographic systems. The number of molecular descriptors that can be obtained for a single analyte is enormous, with some software capable of calculating up to 5000 descriptors per analyte [42]. Such a significant increase in the dimensionality of the descriptors and the incorporation of some nonempirical features could affect the performance of the various QSRR models. Consequently, feature selection methods (variable selection) are necessary to untangle this problem and determine which descriptors are important regarding the retention of the compounds of interest. These methods range from classical types like forward selection and backward elimination to advanced nature-inspired ones like particle swarm optimization (PSO), genetic algorithm (GA) and its descendants (firefly, flower pollination, grasshopper, and ant colony algorithms) [43–52].

Furthermore, various chemometric and machine learning algorithms, such as partial least squares (PLS), multiple linear regression (MLR), artificial neural networks (ANNs), and support vector regression (SVR) were proven to be effective in building reliable QSAR and QSRR models due to their ability in extracting the maximal chemical information while also capturing the possible relationship between the chemical structure and the target property of interest [53–55]. The application of QSRR models has been documented to various chemical families on reversed-phase liquid chromatography, such as non-steroidal anti-inflammatory drugs [56], azole antifungal agents [57], and some analgesics [58].

Support vector regression (SVR), a machine learning algorithm, was first reported by Vapnik, Chervonenkis, and colleagues [59]. The algorithm is based on identifying a linear function that explains most of the variation in the response and simultaneously links the nonlinear relationship between the input and the target data [60]. Compared to conventional regression and neural network algorithms, SVR has some advantages, including good generalization ability, global optimization, and dimensional independence [61]. Because of its capability to model possible nonlinear relationships between molecular descriptors and retention time, it has been incorporated into developing powerful QSRR models [62, 63].

QSRR models could be classified into local models and universal models where local models focus on a specific class of chemical compounds, whereas universal models handle diverse classes in the chemical space. The specificity of the local models makes it perform superior relative to the performance of universal models which characterized by generality [64, 65].

Previously, our group developed two QSRR models that captured the essence of some β-lactam antibiotics retention behavior using MLR models combined with the forward or firefly variable selection algorithms [55]. In our pursuit of studying the QSRR modeling of antibacterial agents, our scope in this work is to investigate the quantitative structure retention relationship in the quinolone and sulfonamide antibacterial classes, highlighting their reversed-phase chromatographic retention mechanisms. Furthermore, investigate the quinolones’ retention behavior with respect to their different ionization states and the organic modifier percentage, as well as the sulfonamides’ retention behavior with respect to the organic modifier percentage. Because of the complexity of the generated data, the use of an advanced variable selection technique coupled with a machine learning algorithm seems imperative. Consequently, the firefly algorithm coupled with SVR was used to develop the target QSRR models. Furthermore, the obtained models were assessed regarding their predictive ability using strict validation criteria; thus, they could be used to predict the retention behavior of potential degradation products and even metabolites of these compounds.

## Experimental

### Solvents, chemicals, sample preparation, and instrumentation

The quinolones (**Fig. S1**) and sulfonamides (**Fig. S2**) under investigation were supplied by different pharmaceutical companies. Pure HPLC-grade acetonitrile, methanol, and dimethylsulfoxide were supplied by Scarlau (Barcelona, Spain). The other chemicals used in this study, including ortho-phosphoric acid, trifluoroacetic acid, sodium dihydrogen orthophosphate, and sodium hydroxide were supplied by Honeywell Riedel-de Haën (Seelze, Germany).

The instruments used in this study included a Jenway 3510, Essex-UK, England pH meter equipped with a glass electrode, and Agilent 1260 HPLC-UV series.

Each drug’s stock solution (2 mg mL^− 1^) was prepared with a suitable solvent either (methanol, dimethylsulfoxide, water, or acetonitrile). These solutions were stored at 4 °C and then diluted with the mobile phase to achieve sample concentrations ranging (0.05–1 mg mL^− 1^) before analysis.

### Chromatographic conditions

The quinolones were eluted chromatographically using an Inertsil^®^ C18 column (250 mm x 4.6 mm, 5 μm) and detected at 275 nm. In a gradient mode, five mobile phases were prepared according to the plan of the experiment, and a chromatographic system was used as programmed in Table 1, using acetonitrile and 28 mM sodium dihydrogen orthophosphate buffer prepared at different pHs 2.2, 3.5, 5.2, 6.5, and 8.2 using ortho-phosphoric acid or sodium hydroxide. However, the pH was measured again after mixing the buffer with acetonitrile and was determined to be 3.2, 4.4, 5.9, 7.32, and 8.9, respectively. The system flow rate was adjusted to 1 ml min^− 1^. After each injection, the system was reconditioned by returning to the initial ratio and remaining constant for 3 min. Data acquisition was performed using the Agilent LC Chemstation software.

**Table 1:** Gradient elution system used in quinolone separation

Time (min)	Acetonitrile %	Buffer %
0	20	80
3	20	80
5	60	40

Sulfonamides were separated chromatographically on a hypersil C18 column (150 mm x 4.6 mm, 5 μm) using isocratic elution based on a mobile phase consisting of acetonitrile and water acidified with trifluoroacetic acid (1 mL. L^− 1^) in different ratios of 50:50, 45:55, or 30:70 v/v and at a flow rate of 0.8 ml min^− 1^. A ratio of 15:85, v/v was initially included but not considered for further assessment because many compounds were strongly retained in the column. The analyses were performed at ambient temperature, with detection at 270 nm. Data acquisition was performed using the Agilent LC Chemstation software.

### QSRR modeling

#### Drawing structures and molecular descriptors calculation and filtration

The major microspecies of the study quinolone at the pH of interest were estimated using the MarvinSketch (6.0.3) [66] generating 21 ions. The canonical smiles of these ions were imported into the Molecular Operating Environment (MOE, 2020.0901) software, where they were converted into 3D structures, and energy was minimized using an RMSD gradient of 0.05 kcal.mol^− 1^Å^−1^ with MMFF94x forcefield. The partial charges were automatically calculated. Finally, MOE molecular mechanical descriptors were computed for all compounds, generating a descriptor fund of 313 descriptors. The initial descriptor fund was reduced by removing zero value and constant descriptors. This resulted in a descriptor fund with 293 descriptors.

In the case of sulfonamides, the PubChem database [67, 68] was used to introduce sulfonamides canonical SMILES into the MOE, where they were converted to 3D structures, and energy was minimized using the same parameters as for quinolones. Afterward, MOE molecular mechanical descriptors were computed for all compounds and a descriptor fund of 313 descriptors was generated. The initial descriptor fund was reduced by removing zero value and constant descriptors, generating a fund of 112 descriptors; moreover, the acetonitrile percentage was incorporated as a descriptor.

#### Training set and test set generation

The 21 quinolones’ major microspecies were divided into a calibration (training) set of 16 molecules and a test set of five molecules. Regarding sulfonamides, a total of 39 experimental retention factors resulted from three different ratios of mobile phase for the 13 compounds that were used in building the QSRR model. The total number of experiments was split into a training set of 30 observations and an external validation test set of nine observations. The selection of the calibration and the validation compounds of quinolones and sulfonamides was based on maintaining the same retention factor value distribution in both sets.

#### Descriptor selection and modeling

Based on Durbin–Watson (DW) test, the linearity of the datasets was tested using augmented partial residual plots (APARP) [69–71]. The test was conducted using a custom script written in MATLAB (R2016 a) [72, 73]. The descriptors that survived the initial filtration were then used to build the QSRR models. The firefly algorithm was implemented in MATLAB and used for descriptor selection as an advanced nature-stimulated algorithm with the RMSE_CV_ of the SVR model serving as the fitness function inside the algorithm for both datasets. The selected descriptors were finally incorporated into the SVR final model building. The algorithm’s parameters were combinatorially optimized such that they were varied in intervals of specific increments, keeping in mind that in all optimization iterations, one parameter was varied while the others remained constant.

#### Model validation

Model validation approaches were performed to evaluate the reliability, robustness, and applicability of the generated models. In the current study, the generated models were validated both internally and externally, and any potential correlation was tested using a Y-scrambling technique, a method commonly used for this purpose.

Internal validation was conducted using leave-one-out cross-validation (CV_LOO_) in the quinolones QSRR model while using leave-10%-out (CV_L10%O_) in the sulfonamides QSRR model. On the other hand, the external validation was conducted by applying the obtained QSRR models to an external validation set of five microspecies of quinolones and nine molecules of sulfonamides. The statistical quality of the models was assessed by calculating the root mean square errors (RMSE) of the prediction and coefficient of the determination.

In Y-randomization validation for the two datasets, the compounds’ output retention factors were shuffled randomly, whereas the compounds’ descriptors remained unscrambled. The resulting datasets were used to build FFA-SVR models using the same protocol as the original models, and the correlation and predictive ability of the resulting models were determined. The entire procedure was repeated 100 times for both datasets.

Hotelling’s T2 and William’s plot methods were used to determine the developed models’ applicability domains (AD) as described in our previous work [55].

## Results and discussion

### Optimization of the FFA and SVR parameters for developing the QSRR models

The firefly algorithm (FFA) was used as a feature selection method to find the relevant descriptors that build reliable QSRR models. The algorithm parameters were initially optimized for proper descriptor selection. Based on our previous study [55], the RMSE_CV_ was used as the fitness function computed by the SVR model to evaluate the models’ performance. A critical parameter in the FFA is the absorption coefficient parameter “γ” because it regulates the light intensity, and thus controls the fireflies’ attractiveness; thus, this parameter has a significant impact on the speed of convergence and the overall behavior of the algorithm. Another valuable parameter is the “α” parameter, which prevents sticking to the local optima by providing some sort of random movements. Finally, the exploration phase of the FFA was controlled by the number of fireflies used, whereas the exploitation phase was controlled by the number of generations. The adjusted FFA parameters obtained through combinatorial optimization are presented in Table 2.

**Table 2:** Parameters of the firefly algorithm used for variable selection in QSRR modeling

Parameter	Quinolones	Sulfonamides
Number of fireflies	10	20
Generations	100	100
α	0.1	0.15
β_ο_	1	1
γ	0.01	0.01

Concerning SVR, different types of kernels as basis function expansions were assessed, including polynomial, radial basis function (RBF), and sigmoid. Initially, the kernel function was examined by evaluating the performance of developed FFA-SVR models, and the RBF was selected as the best kernel function to model the nonlinearity of the generated data. The RBF kernel parameter regulates the amplitude of the Gaussian function and influences the SVR’s generalization ability. Furthermore, two parameters determining the quality of the SVR model were optimized: the penalty error (C), a parameter that controls the trade-off between the complexity of the decision rule and the frequency of error, and the insensitive loss function (ɛ), a precision factor expressing the radius of the tube placed around the regression function f(x). To optimize these parameters, their values were systematically varied in the training step via (CV_LOO_) and (CV_L10%O_) for quinolones and sulfonamides, respectively, while the models’ RMSE_cv_ was monitored. To obtain the optimal ɛ, the SVR with different ɛ values was trained; initially, the value of C was set to 1, but after finding the optimal value of ɛ, the C value was further optimized. It was found that the best models were obtained using kernel types of (RBF), C = 1 and ɛ = 0.01 for both datasets. The final developed FFA-SVR models were used to predict the retention factors of molecules in the test set for quinolones and sulfonamides, respectively.

### QSRR modeling of quinolones in their different ionization states

To elucidate the chromatographic behavior of the quinolones studied, it is important to first understand the relationship between the mobile phase pH and the ionization states of each compound (**Fig. S3**). Some compounds behave ideally with respect to their ionization state, for example, moxifloxacin exists as a cation (polar) at acidic pHs (2.2 and 3.5) but as a neutral compound (hydrophobic) at basic pH (6.5 and 8.2), rationalizing its longer retention factor in basic pH than an acidic one. Ciprofloxacin, lomefloxacin, and norfloxacin exist in different ionization states at pHs (5.2 and 8.2) and this justifies the fluctuation in their retention factors over these pHs. Nadifloxacin exists as a neutral compound at acidic pHs (2.2, 3.5, and 5.2), which explains its longer retention factor at these lower pH values, whereas at basic pH 8.2, it exists as an anionic compound, resulting in rapid elution and a lower retention factor. On the other hand, ofloxacin and danofloxacin exhibit distinct behavior, with their cationic forms appearing at acidic pHs (2.2 and 3.5) exhibiting lower retention factors, whereas their anionic forms present at basic pHs (6.5 and 8.2) exhibit higher retention factors. Additionally, gatifloxacin and gemifloxacin show stability in their retention factors although they can exist in different ionization states across the pH range (2.2–8.2). The calculated retention factors of the eluted quinolones are presented in Table 3. (The raw retention times ± SD are listed in **Table S1** in the supporting material.)

**Table 3:** List of quinolones’ chromatographic retention factors (k)*

Compound name	pH 2.2	pH 3.5	pH 5.2	pH 6.5	pH 8.2
**Gatifloxacin**	1.580	1.603	1.576	1.566	1.635
**Lomefloxacin**	1.405	1.560	1.501	0.920	1.035
**Moxifloxacin**	1.558	1.606	1.592	1.749	1.840
**Nadifloxacin**	2.121	2.148	2.114	2.036	1.685
**Norfloxacin**	1.191	1.162	1.192	1.158	0.384
**Ofloxacin**	1.032	1.176	1.559	1.836	1.885
**Ciprofloxacin**	1.142	1.363	1.294	1.153	0.646
**Gemifloxacin**	1.557	1.576	1.578	1.584	1.633
**Enrofloxacin**	1.622	1.567	1.591	2.468	1.973
**Danofloxacin**	1.370	1.551	1.572	1.822	1.668
**Sparfloxacin**	1.560	1.576	1.567	2.486	1.987

*Dead time = 2.9 min

Based on these previous observations, the behavior of quinolone compounds cannot be predicted solely on their ionization state, and a more in-depth analysis must successfully predict their behavior. It is worth noting that, at a specific pH, a compound can exist in various ionization states and percentages, making it difficult to predict the retention behavior based on single microspecies. To address this issue, we attempted to select the major microspecies as a representative for each molecule in the given pH while avoiding selecting the same microspecies at different pH or retention factors for the same ionization state. Considering this approach, we would be able to derive a simple, interpretable QSRR model that can predict the retention factors of quinolones in their various ionization states.

The first step for quinolones’ QSRR model generation was to check the linearity of the data. Consequently, augmented partial residual plots (APARP) and DW test were used to examine the residuals’ correlation [69–71]. The associated probability was found to be 0.045 (< 0.05) indicating the significance of the test and nonlinearity of the data; thus, nonlinear models such as ANN and SVR were tried for data modeling, with SVR yielding the best results.

Five descriptors were chosen by the FFA and combined in building the SVR model (SMR, GCUT_SLOGP_1, VSA, Vsurf_EWmin 2, and Vsurf_IW6). SMR is a 2D descriptor linked to molecular refractivity, which includes implicit hydrogens [74]. This property is an atomic contribution model that assumes the correct protonation state. GCUT_SLOGP_1 is a 2D descriptor that uses atomic contribution to logP in place of partial charge. VSA is a 3D descriptor related to the surface area, volume, and shape of molecules; it represents van der Waals’ surface area [75]. Vsurf_EWmin 2 is a 3D descriptor that represents the second lowest hydrophilic energy. Vsurf_IW6 is a 3D descriptor that represents the hydrophilic integy moment at (− 4.0). Considering the selected descriptors, the model displays that quinolones retention depends on their size and hydrophobic/hydrophilic nature, which is consistent with the main elements influencing the retention in reversed-phase liquid chromatography.

**Fig. 1:**
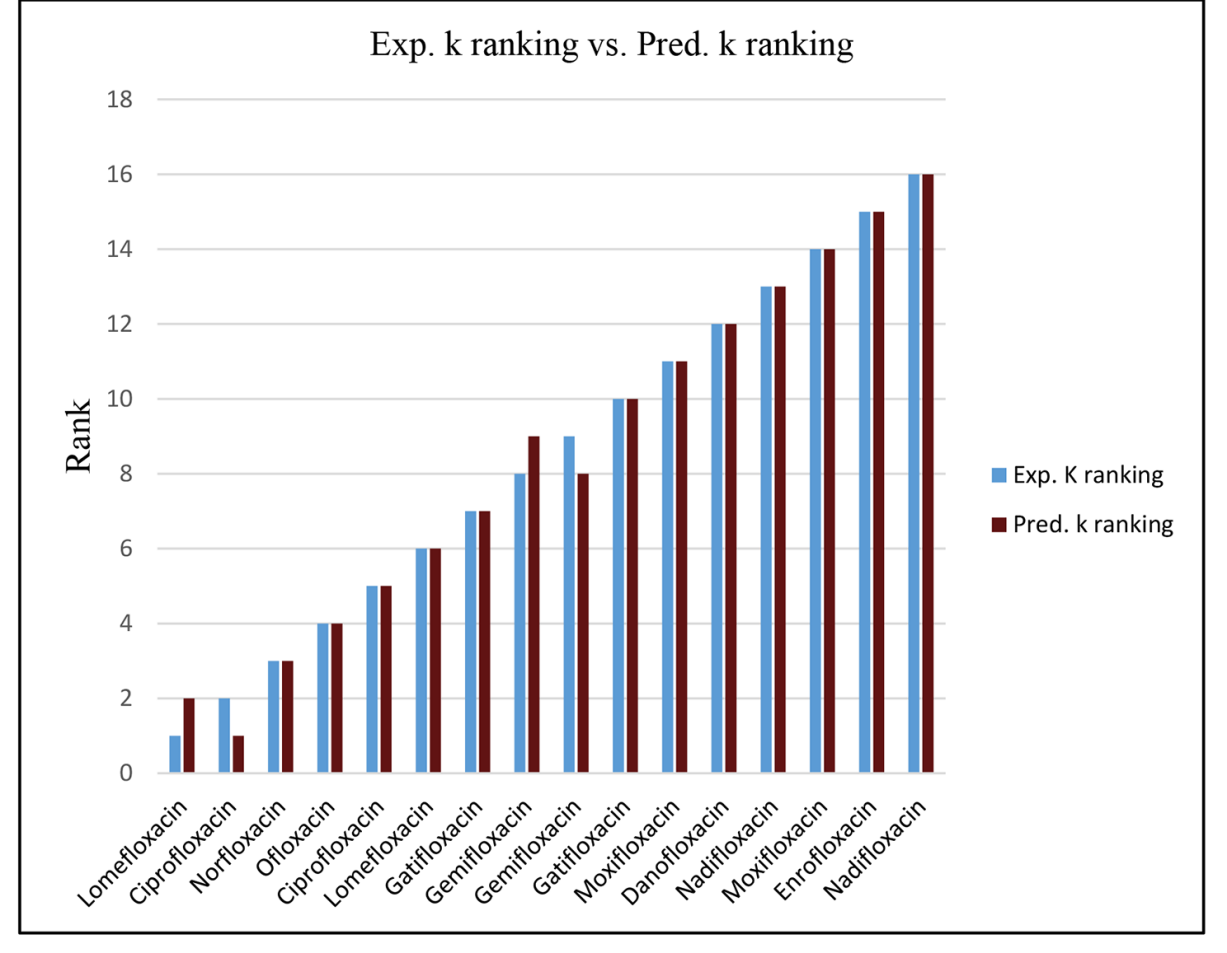
FFA-SVR model experimental k ranking vs. predicted k ranking in quinolone training set prediction

Regarding the performance of the developed QSRR model for the quinolones, the agreement of the experimental and predicted retention factors demonstrates the model’s good predictive capability, as shown in Table 4. The proximity between the training set prediction and the cross-validation results indicates the robustness of the resulting QSRR model and its lack of any overfitting. As shown in Table 5, the results demonstrate the good prediction capability of the obtained model. The correlation between the experimental and predicted retention factors for the training set, test set, and CV_LOO_ results are presented in the supporting materials (**Figs. S4 and S5**). The Spearman ranking correlation coefficient (ρ) was also calculated and found to be 0.976, 0.982, and 0.900 for the training set prediction (ρ_cal_), CV _LOO_ (ρ_LOO_), and the external test set (ρ_pred_), respectively, (Table 5). The closeness of ρ to “1” indicates a reasonable accuracy and excellent capability of the generated model to reproduce the experimental retention factor ranking (Fig. 1).

**Table 4:** Experimental and predicted retention factors (k) of quinolone compounds in the training set, cross-validation, and test set prediction

Compound name	Buffer pH	Experimental k	Training set prediction	Residuals	Cross-Validation CV_LOO_	Residuals
**Lomefloxacin**	6.5	0.920	1.175	0.255	1.247	0.327
**Ciprofloxacin**	6.5	1.153	1.164	0.011	1.262	0.109
**Norfloxacin**	3.5	1.162	1.255	0.093	1.342	0.18
**Ofloxacin**	3.5	1.176	1.261	0.085	1.385	0.209
**Ciprofloxacin**	3.5	1.363	1.330	−0.033	1.293	−0.07
**Lomefloxacin**	3.5	1.560	1.550	−0.01	1.401	−0.159
**Gatifloxacin**	6.5	1.566	1.555	−0.011	1.493	−0.073
**Gemifloxacin**	3.5	1.576	1.586	0.01	1.593	0.017
**Gemifloxacin**	6.5	1.584	1.575	−0.009	1.558	−0.026
**Gatifloxacin**	3.5	1.603	1.613	0.01	1.621	0.018
**Moxifloxacin**	3.5	1.606	1.616	0.01	1.638	0.032
**Danofloxacin**	8.2	1.668	1.659	−0.009	1.662	−0.006
**Nadifloxacin**	8.2	1.685	1.694	0.009	1.763	0.078
**Moxifloxacin**	6.5	1.749	1.739	−0.01	1.700	−0.049
**Enrofloxacin**	8.2	1.973	1.819	−0.154	1.773	−0.2
**Nadifloxacin**	3.5	2.148	1.828	−0.32	1.769	−0.379
Norfloxacin*	6.5	1.158	1.149	−0.009		
Danofloxacin*	3.5	1.551	1.433	−0.118		
Sparfloxacin*	3.5	1.576	1.563	−0.013		
Enrofloxacin*	2.2	1.622	1.493	−0.129		
Ofloxacin*	8.2	1.885	1.603	−0.282		

(*) Test set compound

**Table 5:** Quinolones and sulfonamides FFA-SVR model performance evaluation parameters

Parameter	Quinolones FFA-SVR	Sulfonamides FFA-SVR
**R** ^**2**^_**cal**_	0.931	0.900
**R** ^**2**^_**cal−adj**_	0.926	0.896
**q** ^**2**^_**LOO**_	0.808	---
**q** ^**2**^_**L10%O**_	---	0.812
**R** ^**2**^_**pred**_	0.879	0.820
**RMSE**_**cal**_	0.114	0.240
**RMSE**_**CVLOO**_	0.163	0.328
**RMSE**_**pred**_	0.148	0.450
**ρ**_**cal**_	0.976	0.988
**ρ**_**Loo**_	0.982	---
**ρ**_**L10%O**_	---	0.941
**ρ**_**pred**_	0.900	0.883

### QSRR modeling of sulfonamides using different organic modifiers

QSRR modeling of sulfonamides was implemented to study the associations between the retention factors of the examined compounds eluted using different percentages of acetonitrile in the mobile phase composition (50%, 45%, and 30%), (See **Fig. S6**), and their calculated constitutional, geometrical, physicochemical, and electronic descriptors (independent variables). The raw retention times ± SD and the calculated retention factors of eluted sulfonamides are shown in **Table S2** and Table 6, respectively.

The linearity of the data was first considered using the same procedures conducted in the quinolone dataset, with an associated probability of 3.2^− 17^ (< 0.05) indicating the nonlinearity of the generated data. The FFA-SVR model was used in this case, resulting in two descriptors plus acetonitrile percentage in building the QSRR model. The selected features (Vsurf-D2 and vsurf-w2) are 3D descriptors related to the molecular hydrophobic and hydrophilic volumes, respectively. The QSRR model indicates that, in addition to the influence of the third descriptor (acetonitrile percentage in the mobile phase), the sulfonamide analytes retention depends on their hydrophobic/hydrophilic nature, which is a common element that plays an important role in the differential elution of analytes in reversed-phase liquid chromatography.

**Table 6:** List of sulfonamides chromatographic retention factors (k) *

Compound name	Acetonitrile%		
	**50%**	**45%**	**30%**
**Sulfacetamide Na**	0.154	0.203	0.393
**Sulfaguanidine**	0.170	0.188	0.256
**Sulfadiazine**	0.174	0.228	0.443
**Sulfaclozine**	0.549	0.752	2.196
**Sulfadimethoxine**	0.419	0.567	1.433
**Sulfadimidine**	0.311	0.389	0.730
**Sulfadoxine**	0.395	0.524	1.276
**Sulfathiazole**	0.166	0.221	0.426
**Sulfachloropyrazine Na**	0.546	0.754	2.177
**Sulfanilamide**	0.154	0.194	0.295
**Sulfamethoxazole**	0.421	0.568	1.548
**Sulfapyridine**	0.306	0.359	0.597
**Sulfaquinoxaline**	0.519	0.716	2.221

*Dead time = 2.0 min

**Fig. 2:**
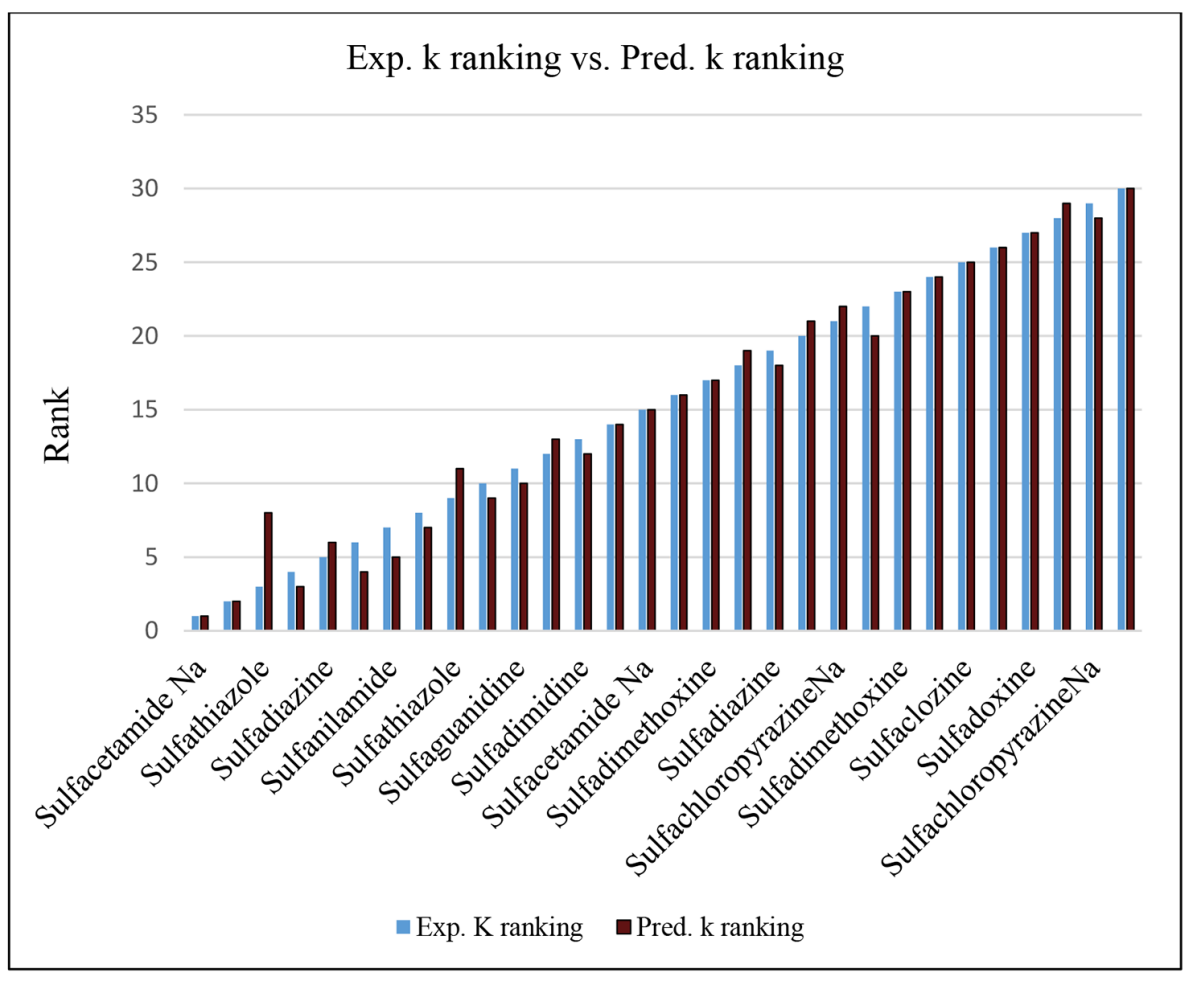
FFA-SVR model experimental k ranking vs. predicted k ranking in Sulfonamides training set prediction

The results also demonstrate the obtained model’s good prediction capability, as shown in Tables 5 and 7. The model training and test set correlation of the experimental and predicted retention are presented in the supporting material (**Fig. S7**), while the compounds’ experimental and predicted retention in the CV _L10%O_ are presented in the supporting material (**Fig. S8**), indicating the good correlation and the generalizability of the developed QSRR sulfonamide model. The Spearman ranking correlation coefficient (ρ) was calculated for the training set prediction (ρ_cal_), CV _L10%O_ (ρ_L10%O_), and the external test set (ρ_pred_) and was found to be 0.988, 0.941, and 0.883, respectively (Fig. 2). The proximity of ρ to “1” indicates the capability of the generated model to reproduce the experimental retention factor ranking of the compounds under investigation with reasonable accuracy.

Furthermore, the residual plots for both classes show the differences between the predicted and the experimental retention factors (residuals) for the various compounds. The random dispersion of the residuals around the horizontal axis confirmed the model’s prediction ability (see supporting materials) (**Figs. S9 and S10**). The prediction accuracy of the generated local focused models is acceptable and comparable to that of the generalized universal models [64, 65].

**Table 7:** Experimental and predicted retention factors (k) of sulfonamide compounds in the training set, cross-validation, and test set

Compound name	Acetonitrile %	Experimental k	Training set prediction	Residuals of Training set	Cross-Validation CV_L10%O_	Residuals of Cross-Validation
**Sulfacetamide Na**	50%	0.154	0.153	−0.001	0.151	−0.002
**Sulfacetamide Na**	45%	0.203	0.194	−0.009	0.171	−0.032
**Sulfacetamide Na**	30%	0.393	0.385	−0.008	0.336	−0.057
**Sulfaguanidine**	50%	0.170	0.178	0.009	0.212	0.042
**Sulfaguanidine**	45%	0.188	0.181	−0.006	0.182	−0.006
**Sulfaguanidine**	30%	0.256	0.267	0.011	0.458	0.202
**Sulfadiazine**	50%	0.174	0.185	0.011	0.193	0.019
**Sulfadiazine**	45%	0.228	0.237	0.010	0.236	0.008
**Sulfadiazine**	30%	0.443	0.454	0.011	0.520	0.078
**Sulfaclozine**	50%	0.549	0.528	−0.021	0.460	−0.088
**Sulfaclozine**	45%	0.752	0.701	−0.051	0.657	−0.095
**Sulfaclozine**	30%	2.196	1.310	−0.886	1.082	−1.114
**Sulfadimethoxine**	50%	0.419	0.429	0.010	0.427	0.008
**Sulfadimethoxine**	45%	0.567	0.576	0.009	0.602	0.035
**Sulfadimethoxine**	30%	1.433	1.278	−0.155	1.006	−0.427
**Sulfadimidine**	50%	0.311	0.299	−0.012	0.289	−0.022
**Sulfadimidine**	45%	0.389	0.378	−0.010	0.352	−0.037
**Sulfadimidine**	30%	0.730	0.643	−0.087	0.555	−0.175
**Sulfadoxine**	50%	0.395	0.405	0.010	0.419	0.024
**Sulfadoxine**	45%	0.524	0.540	0.017	0.569	0.046
**Sulfadoxine**	30%	1.276	1.211	−0.065	0.962	−0.314
**Sulfathiazole**	50%	0.166	0.224	0.058	0.242	0.076
**Sulfathiazole**	45%	0.221	0.285	0.064	0.289	0.068
**Sulfathiazole**	30%	0.426	0.521	0.095	0.574	0.148
**Sulfachloropyrazine Na**	50%	0.546	0.568	0.022	0.623	0.078
**Sulfachloropyrazine Na**	45%	0.754	0.742	−0.012	0.698	−0.056
**Sulfachloropyrazine Na**	30%	2.177	1.233	−0.944	0.937	−1.240
**Sulfanilamide**	50%	0.154	0.165	0.011	0.231	0.078
**Sulfanilamide**	45%	0.194	0.183	−0.010	0.167	−0.026
**Sulfanilamide**	30%	0.295	0.307	0.012	0.409	0.114
**Sulfacetamide Na**	50%	0.154	0.153	−0.001	0.151	−0.002
**Sulfacetamide Na**	45%	0.203	0.194	−0.009	0.171	−0.032
**Sulfacetamide Na**	30%	0.393	0.385	−0.008	0.336	−0.057
**Sulfaguanidine**	50%	0.170	0.178	0.009	0.212	0.042
**Sulfaguanidine**	45%	0.188	0.181	−0.006	0.182	−0.006
**Sulfaguanidine**	30%	0.256	0.267	0.011	0.458	0.202
**Sulfadiazine**	50%	0.174	0.185	0.011	0.193	0.019
**Sulfadiazine**	45%	0.228	0.237	0.010	0.236	0.008
**Sulfadiazine**	30%	0.443	0.454	0.011	0.520	0.078
**Sulfaclozine**	50%	0.549	0.528	−0.021	0.460	−0.088
**Sulfaclozine**	45%	0.752	0.701	−0.051	0.657	−0.095
**Sulfaclozine**	30%	2.196	1.310	−0.886	1.082	−1.114
**Sulfadimethoxine**	50%	0.419	0.429	0.010	0.427	0.008
**Sulfadimethoxine**	45%	0.567	0.576	0.009	0.602	0.035
**Sulfadimethoxine**	30%	1.433	1.278	−0.155	1.006	−0.427
Sulfamethoxazole*	50%	0.421	0.248			
Sulfamethoxazole*	45%	0.568	0.322			
Sulfamethoxazole*	30%	1.548	0.644			
Sulfapyridine*	50%	0.306	0.283			
Sulfapyridine*	45%	0.359	0.306			
Sulfapyridine*	30%	0.597	0.443			
Sulfaquinoxaline*	50%	0.519	0.530			
Sulfaquinoxaline*	45%	0.716	0.719			
Sulfaquinoxaline*	30%	2.221	1.279			

-Test set compound (*)

### Y-scrambling validation

Y-randomization or permutation test is another criterion used to validate our findings in this study, especially with this small number of observations, to ensure that the obtained models are due to a true correlation between the selected descriptors and the target retention factors rather than statistical chance. It is suspected that the original QSRR model is significant if there is a solid link between the selected descriptors and the original response variables. Y-randomization was repeated 100 times, if the statistical attributes of these randomized models are significantly lower than the original one, it can be concluded that the model is sensible and was not obtained by chance. The equation below was used to evaluate the quality of the obtained models from the 100 randomized matrices and to compare it with the original model quality. ^c^R_p_^2^ should be above 0.5 to ensure that the original model is not obtained by chance [76].

$${{\text{c}}_{\text{R}}}_{\text{p}}^{2}=\text{R}\text{*} \sqrt{{\text{R}}^{2}-{\text{R}}_{\text{y}}^{2}}$$

Where (^c^R_p_^2^) is the degree of variation in the values of the squared correlation coefficient average of the randomized models R_y_^2^ and the squared correlation coefficient of the original model R^2^.

**Fig. 3:**
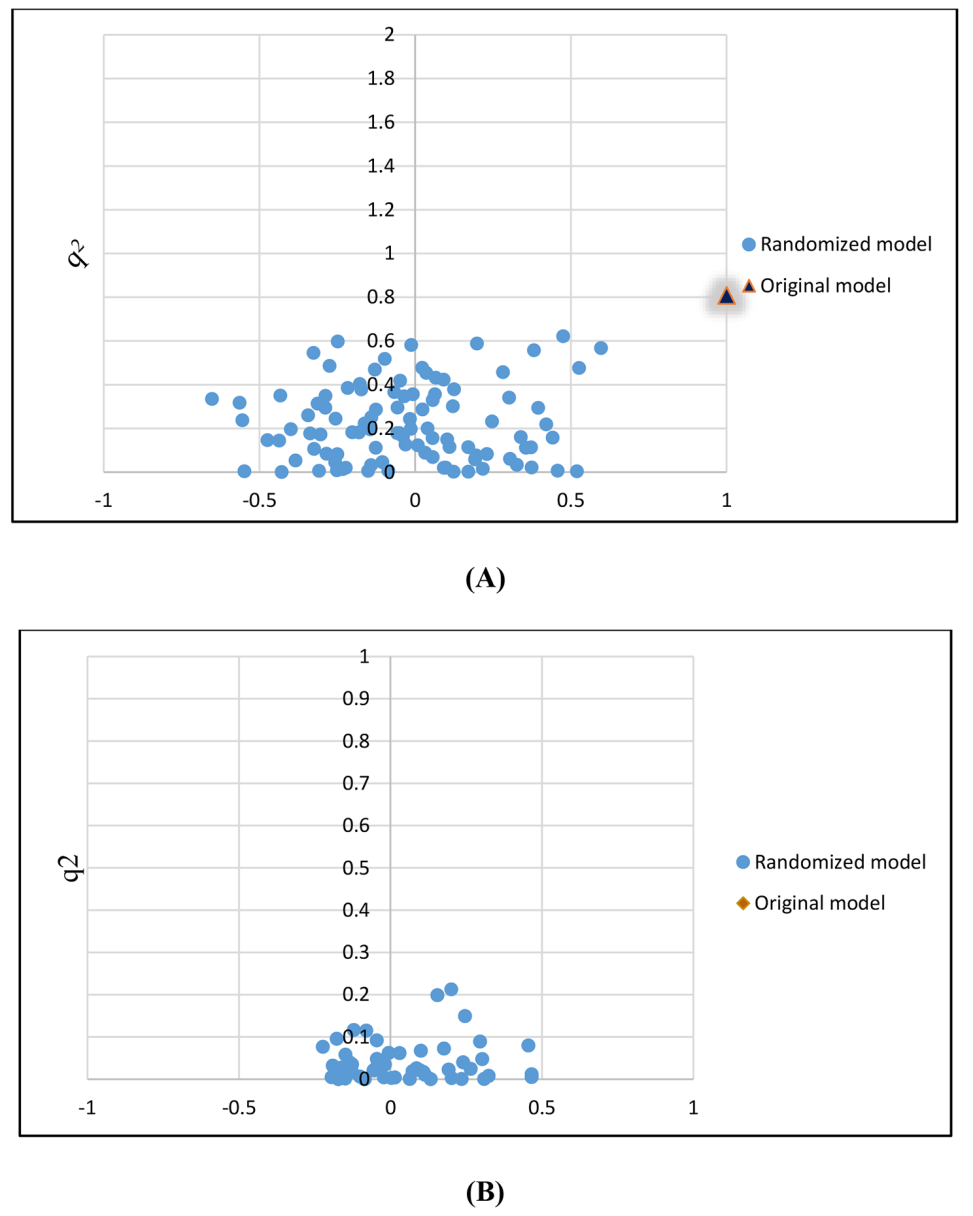
Y-randomization validation results for the FFA-SVR for (A) quinolone and (B) sulfonamide modeling

The statistical parameters of the scrambled models gathered around zero in a symmetrical pattern for both data (Fig. 3**)**, indicating that the scrambled models are of an extremely low quality. ^c^Rp^2^ values calculated for cross-validation were found to be 0.687 and 0.791 (more than 0.5) for quinolones and sulfonamides QSRR models, respectively, which negates that the obtained model is the result of a chance correlation.

### Applicability domain of both QSRR models

**Fig. 4:**
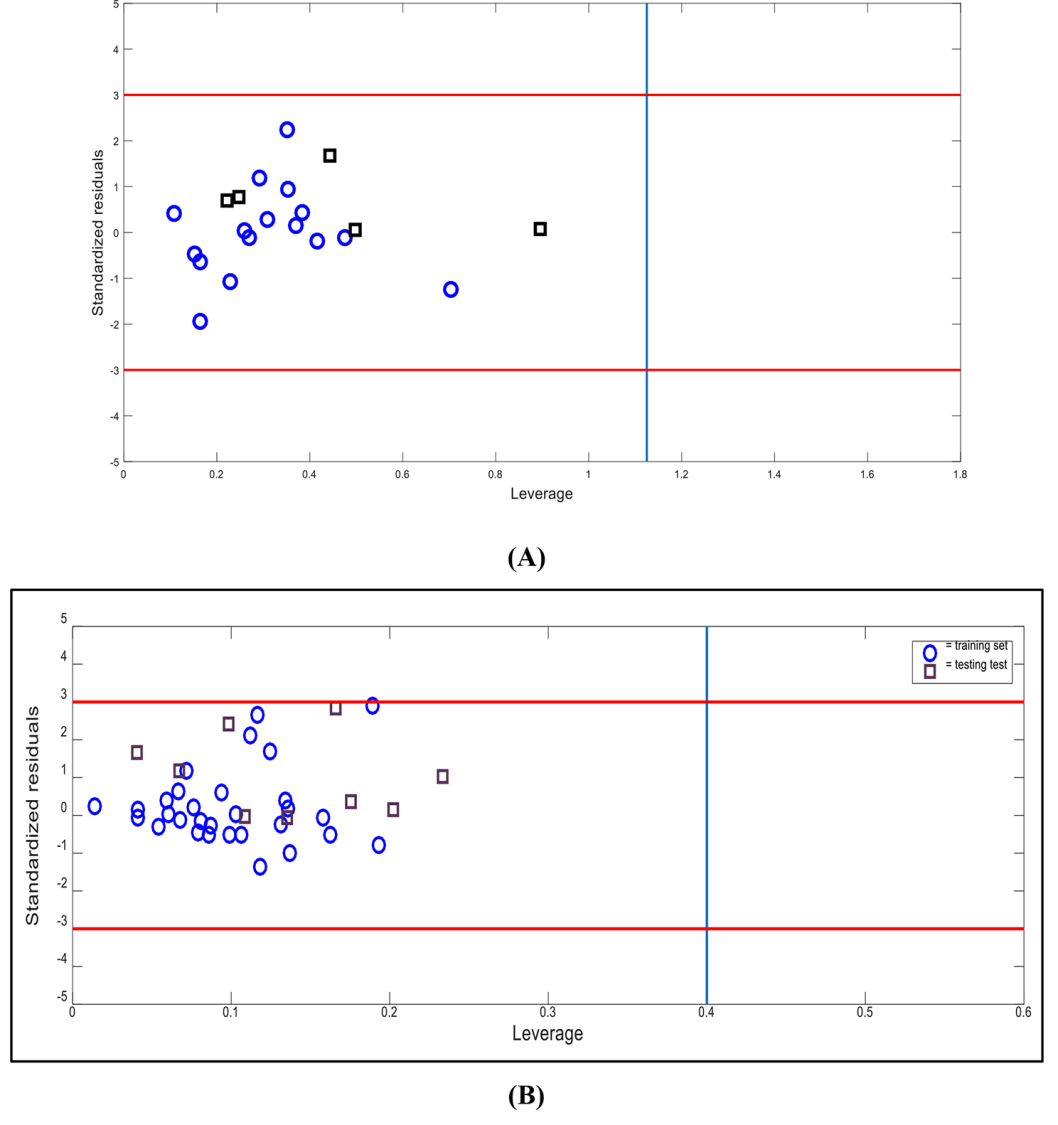
Williams plot for FFA-SVR models of (**A**) quinolones with ± 3 standard deviations, and a leverage threshold h* of 1.125 as warning limits, and (**B**) sulfonamides with ± 3 standard deviations, and a leverage threshold h* of 0.4 as warning limits. Circles represent the training set cross-validation prediction and diamonds represent the test set prediction.

The applicability domain of a QSPR is the structural, biological space, or physicochemical knowledge or information on which the model’s training set was developed and for which it is applicable to make predictions for new compounds. In William’s plot for the FFA-SVR models, the applicability domain is inside a squared area within ± 3 standard deviations and has a leverage threshold h* of 1.125 and 0.4 for quinolones and sulfonamides, respectively. The prediction is only considered reliable for those compounds that fall within this AD. It can be seen that all compounds (training and test sets) fall within this range, with no outliers (Fig. 4).

## Conclusion

Two QSRR models were generated for predicting the retention behavior of quinolones and sulfonamides in the HPLC system. The influence of the pH of the mobile phase on the ionization state and hence the retention factor of each quinolone, as well as the effect of acetonitrile composition in the mobile phase on the retention factors of sulfonamides, were investigated, resulting in the selection of 21 major microspecies of quinolones and 39 sulfonamide compounds. In both classes, significant descriptors related to retention behavior in the chromatographic system were selected using the advanced FFA and then incorporated into building the QSRR models using the SVR algorithm. The two models performed well on both the training and the validation levels. In quinolones, the regression coefficients of the training set prediction (R^2^_cal_), CV _LOO_ (q^2^_LOO_), and the external test set (R^2^_pred_) were 0.931 (R^2^_adjusted_ = 0.926), 0.808, and 0.879, respectively, with RMSE of 0.114, 0.163, and 0.148, respectively. In sulfonamides, the regression coefficients of the training set prediction (R^2^_cal_), CV _L10%O_ (q^2^_L10%O_) and the external test set (R^2^_pred_) were 0.900 (R^2^_adjusted_ = 0.896), 0.812 and 0.820, respectively, with RMSE of 0.240, 0.450, and 0.328, respectively. In the Y-randomization validation test, the two models had ^c^R_p_^2^ values of 0.687 and 0.791 for quinolones and sulfonamides, respectively, indicating that both models are significant and were not obtained by chance.

## Electronic supplementary material

Below is the link to the electronic supplementary material.

Supplementary Material 1

Supplementary Material 2

Supplementary Material 3

## Data Availability

All data generated or analyzed during this study are included in this published article and its supplementary information files.
