# Supplementary material for: QSRR modeling of the chromatographic retention behavior of some quinolone and sulfonamide antibacterial agents using firefly algorithm coupled to support vector machine
Source: BMC Chem. 2022 Nov 3;16(1):85. doi: 10.1186/s13065-022-00874-2 (PMC9635186; doi:10.1186/s13065-022-00874-2)
Supplement: Supplementary file 1 — Supplementary Material 1 [file 13065_2022_874_MOESM1_ESM.docx]

**QSRR modeling of the chromatographic retention behavior of some quinolone and sulfonamide antibacterial agents using firefly algorithm coupled to support vector machine**

Marwa A. Fouad^a,b*^, Ahmed Serag^c^, Enas H. Tolba^d^, Manal A. El-Shal^d^ and Ahmed M. El Kerdawy^a^

^a^ Pharmaceutical Chemistry Department, Faculty of Pharmacy, Cairo University, Kasr El-Aini St., Cairo, P.O. Box 11562, Egypt

^b^ Department of Pharmaceutical Chemistry, School of Pharmacy, Newgiza University (NGU), Newgiza, km 22

Cairo–Alexandria Desert Road, Cairo, Egypt

^c^ Pharmaceutical Analytical Chemistry Department, Faculty of Pharmacy, Al-Azhar University, Cairo 11751, Egypt

^d^ Egyptian Drug Authority (Former National Organization for Drug Control and Research), Cairo, Egypt

**Fig. S1:** Chemical structures of the quinolones under investigation

**Fig. S2:** Chemical structures of the sulfonamides under investigation


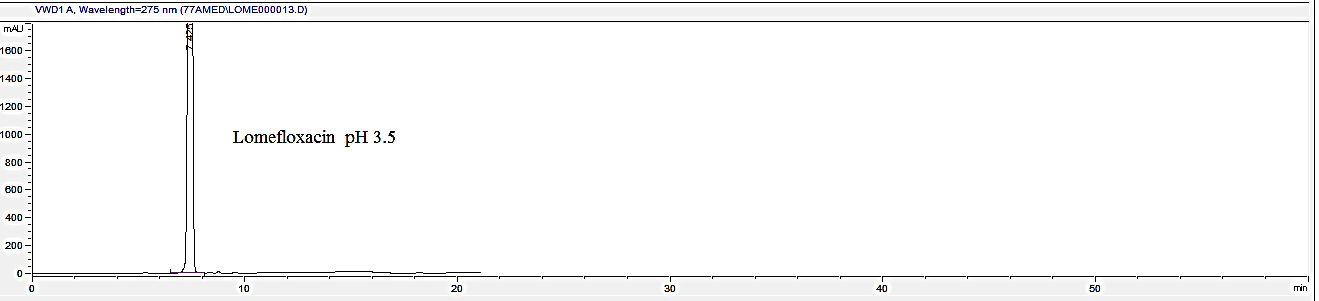


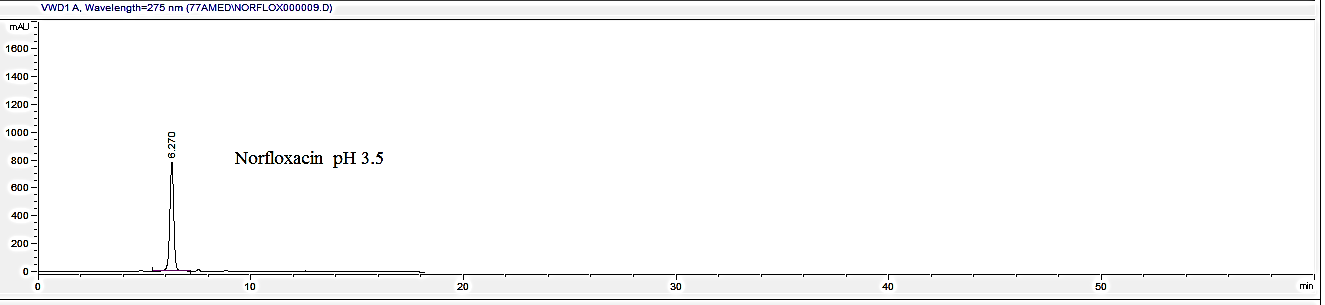


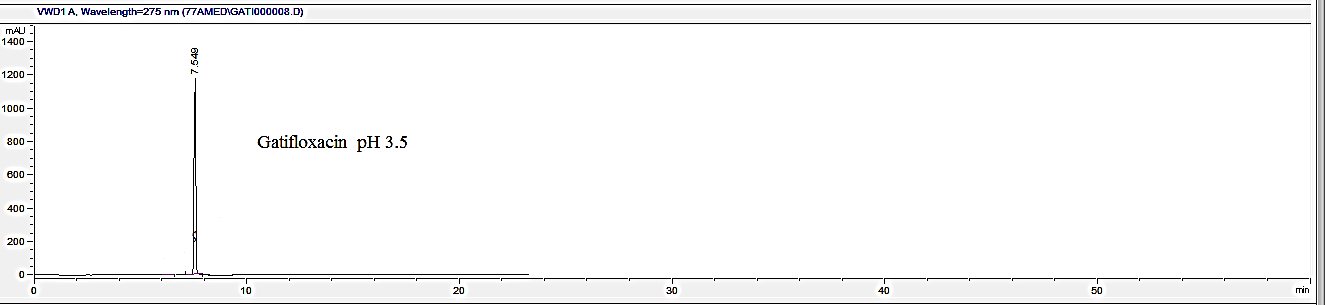


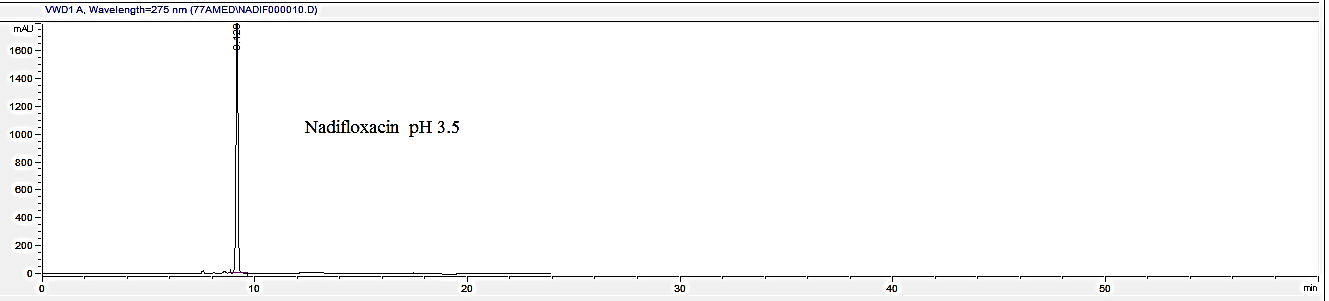


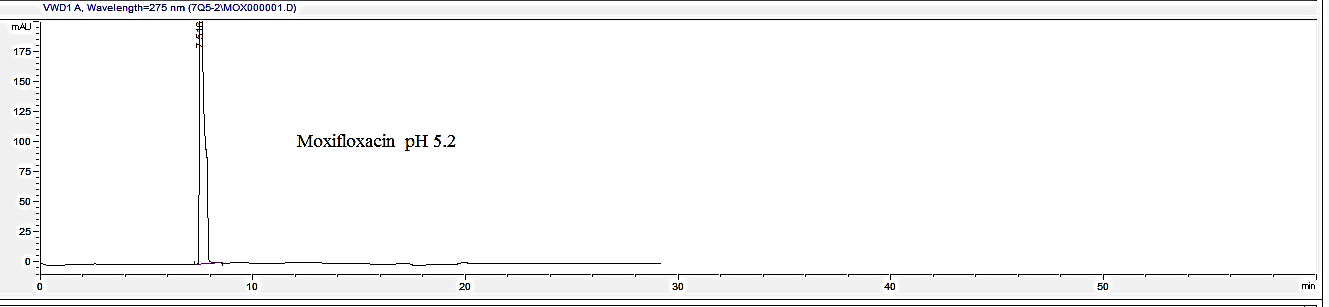


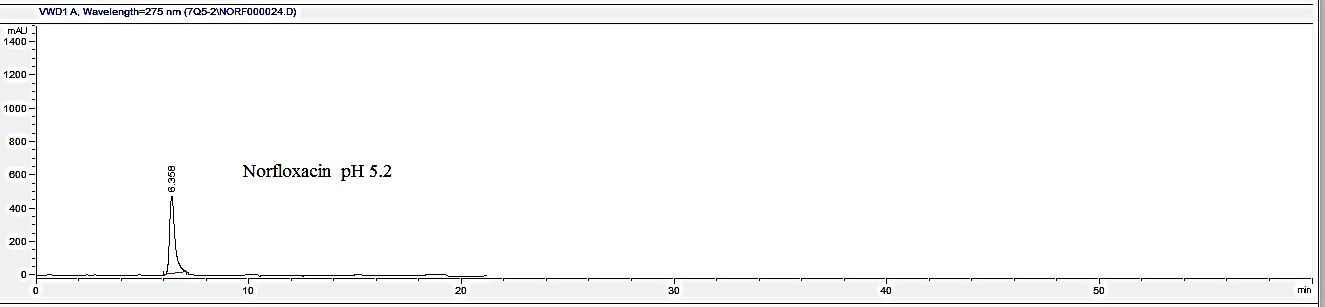


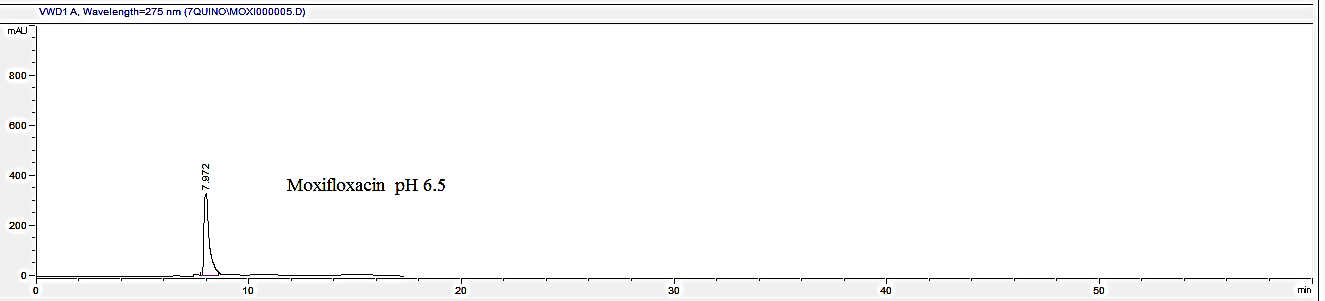


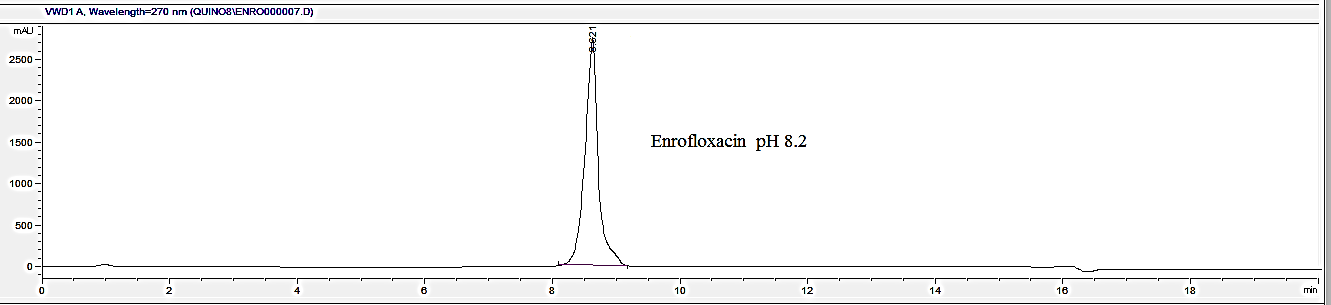


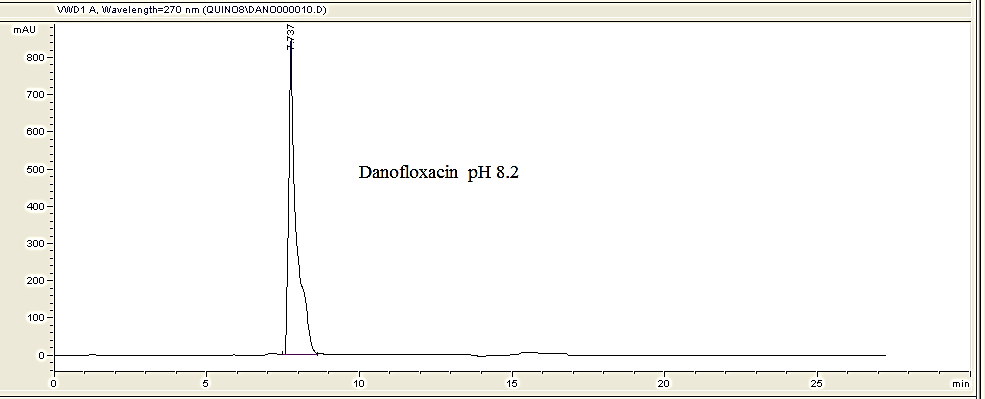


**Fig. S3:** Representative chromatograms of different quinolones eluted at different pHs.

**Fig. S4:** Training and test set predictive ability of FFA-SVM for quinolones modeling.

**Fig. S5:** Cross validation predictive ability of FFA-SVM for quinolones modeling.

Ratio (50:50) v/v

Ratio (50:50) v/v

Ratio (50:50) v/v

Ratio (50:50) v/v

Ratio (50:50) v/v

Ratio (45:55) v/v

Ratio (45:55) v/v

Ratio (45:55) v/v

Ratio (45:55) v/v

Ratio (30:70) v/v

Ratio (30:70) v/v

Ratio (30:70) v/v

Ratio (30:70) v/v

Ratio (30:70) v/v

Ratio (30:70) v/v

Ratio (30:70) v/v

# **Fig. S6:** Chromatograms of different sulfonamides eluted at different ratios of acetonitrile and water acidified with trifluoroacetic acid.

**Fig. S7:** Training and test set predictive ability of sulfonamides FFA-SVM model.

**Fig. S8:** Cross validation predictive ability of sulfonamides FFA-SVM model.

**Fig. S9:** Residual plot for quinolones QSRR model in the training set prediction.

**Fig. S10:** Residual plot for sulfonamides QSRR model in the training set prediction.

**Table (S1):** List of quinolones chromatographic retention times ± SD

| **Compound name** | **pH 2.2** | **pH 3.5** | **pH 5.2** | **pH 6.5** | **pH 8.2** |
| --- | --- | --- | --- | --- | --- |
| **Gatifloxacin** | 7.48±0.131 | 7.55±0.134 | 7.47±0.105 | 7.44±0.089 | 7.64±0.137 |
| **Lomefloxacin** | 6.98±0.046 | 7.43±0.105 | 7.25±0.05 | 5.57±0.099 | 5.9±0.043 |
| **Moxifloxacin** | 7.42±0.115 | 7.56±0.089 | 7.52±0.105 | 7.97±0.071 | 8.24±0.105 |
| **Nadifloxacin** | 9.05±0.054 | 9.13±0.074 | 9.03±0.118 | 8.8±0.141 | 7.79±0.131 |
| **Norfloxacin** | 6.36±0.069 | 6.27±0.091 | 6.36±0.054 | 6.26±0.146 | 4.01±0.075 |
| **Ofloxacin** | 6.19±0.128 | 6.79±0.049 | 7.48±0.15 | 9.2±0.055 | 7.6±0.145 |
| **Ciprofloxacin** | 5.89±0.102 | 6.31±0.091 | 7.42±0.046 | 8.22±0.116 | 8.37±0.064 |
| **Gemifloxacin** | 6.21±0.096 | 6.85±0.073 | 6.65±0.048 | 6.24±0.083 | 4.77±0.058 |
| **Enrofloxacin** | 7.42±0.14 | 7.47±0.112 | 7.48±0.082 | 7.5±0.114 | 7.64±0.1 |
| **Danofloxacin** | 10.02±0.049 | 10.2±0.137 | 10.29±0.05 | 9.48±0.047 | 3.9±0.077 |
| **Sparfloxacin** | 7.6±0.142 | 7.44±0.08 | 7.51±0.134 | 10.06±0.056 | 8.62±0.076 |

**Table (S2):** List of sulfonamides chromatographic retention times ± SD.

| **Compound name** | **Acetonitrile%** | | |
| --- | --- | --- | --- |
|  | **50%** | **45%** | **30%** |
| **Sulfacetamide Na** | 2.31±0.053 | 2.41±0.082 | 2.79±0.088 |
| **Sulfaguanidine** | 2.34±0.077 | 2.38±0.029 | 2.51±0.04 |
| **Sulfadiazine** | 2.35±0.074 | 2.46±0.048 | 2.89±0.067 |
| **Sulfaclozine** | 3.1±0.044 | 3.5±0.039 | 6.39±0.031 |
| **Sulfadimethoxine** | 2.84±0.081 | 3.13±0.071 | 4.87±0.057 |
| **Sulfadimidine** | 2.62±0.045 | 2.78±0.076 | 3.46±0.073 |
| **Sulfadoxine** | 2.79±0.031 | 3.05±0.043 | 4.55±0.082 |
| **Sulfathiazole** | 2.33±0.021 | 2.44±0.077 | 2.85±0.057 |
| **Sulfachloropyrazine Na** | 3.09±0.037 | 3.51±0.088 | 6.35±0.031 |
| **Sulfanilamide** | 2.31±0.061 | 2.39±0.033 | 2.59±0.059 |
| **Sulfamethoxazole** | 2.84±0.048 | 3.14±0.055 | 5.1±0.023 |
| **Sulfapyridine** | 2.61±0.067 | 2.72±0.056 | 3.19±0.049 |
| **Sulfaquinoxaline** | 3.04±0.054 | 3.43±0.038 | 6.44±0.033 |

**Description of the provided excel files:**

**Quinolone Dataset File:**

**Sheet A:** Calculated descriptors for the studied quinolones using MOE.

**Sheet B:** The calculated quinolones descriptors after constant removal filtration.

**Sheet C:** The calculated quinolones descriptors after filtration based on standard deviation.

**Sheet D:** Training set of the studied quinolones used in the QSRR model.

**Sheet E:** Test set of the studied quinolones used in the QSRR model.

**Sheet F:** Validation results of the scrambled quinolone QSRR model.

**Sulfonamide Dataset File:**

**Sheet A:** Calculated descriptors for the studied sulfonamides using MOE.

**Sheet B:** The calculated sulfonamides descriptors after constant removal and standard deviation filtration.

**Sheet C:** Training set and test set of the studied sulfonamides used in the QSRR model.

**Sheet D:** Retention times and retention factors of the sulfonamides compounds.
